# Color naming in Tsimane’–Spanish bilinguals indicates that differential experience with content domains affects lexical access

**DOI:** 10.1038/s41598-022-18461-9

**Published:** 2022-10-19

**Authors:** Saima Malik-Moraleda, Manuel Roca, Edward Gibson

**Affiliations:** 1 Department of Brain and Cognitive Sciences, Massachusetts Institute of Technology, Cambridge, MA 02139 USA; 2 Program in Speech and Hearing Bioscience and Technology, Harvard University, Boston, MA 02114 USA; 3 McGovern Institute for Brain Research, Massachusetts Institute of Technology, Cambridge, MA 02139 USA; 4 Centro Boliviano de Dessarrollo Socio Integral, San Borja, Beni Bolivia

**Keywords:** Psychology, Human behaviour

## Abstract

A standard assumption in the bilingual language processing literature is that the ease of access of a word in a language is determined by the speaker’s *overall* proficiency in the language. Alternatively, it could be that proficiency varies across semantic categories of the bilingual’s two languages. Here, we investigated lexical access in color terms in Tsimane’–Spanish bilinguals. Given that color terms are generally more frequent in Spanish than Tsimane’, participants may have better lexical access for color words in Spanish despite being overall more proficient in Tsimane’. Twenty-two Tsimane’–Spanish bilinguals took part in a picture naming task where participants labeled colors and animals. Participants were equally fast and accurate at naming animals in Tsimane’ and Spanish. However, participants were faster and more accurate at naming colors in Spanish than Tsimane’ except for the three color words that are most frequent (*jaibes* ~ white, *tsincus* ~ black, *jaines* ~ red) in Tsimane’, for which they were equally fast in both Tsimane’ and Spanish. These results suggest that category-specific proficiency is a better predictor for lexical access than overall proficiency.

## Introduction

Humans express their thoughts and ideas one word a time. How do speakers choose which word to produce next? This question is rendered more complex for the majority of the world’s population, who speak more than one language^1–3^, as they not only have to produce the right word, but have to produce it in the right language^4–6^. While bilingualism is a heterogeneous phenomenon^7–10^, in general, bilingual speakers report being better at naming words in the language they acquired earlier and consider themselves to be more proficient in^11–13^. The idea seems deceptively simple: the better you are *overall* in a language, the better you are at naming individual words in that language. However, it is possible that *overall* proficiency in a language is not similar across all categories of that language. For instance, a non-native English speaker that attends an English-based university might be more proficient for those academic terms in English than their native language, despite being overall more proficient in their native language^14^. Yet, most research in bilingualism tends to assess and report overall proficiency, assuming it to be similar across different categories in the language.

Do models of bilingual lexical access distinguish between overall proficiency (i.e., the ability to speak fluently in a language) and category-specific proficiency (i.e., the ability to speak fluently about certain word categories, such as academic terminology in the example above)? According to the earliest proposed model of bilingual lexical access, lexical selection occurs only within the target language, thus predicting that words in the non-target language will never be activated regardless of frequency and proficiency of the speaker^15^. However, this model is not supported by experimental evidence showing that words in both languages are activated even when only one is intended to be produced^16^.

Thus Costa et al. proposed a second model, the language-specific selection model, according to which, lexical candidates in both languages are activated during production, and the target language restricts selection to words in that language. This model accounts for the activation of both languages even when only one is intended to be produced as follows: an independent semantic system is shared between the two languages, and relevant semantic information is activated regardless of the language spoken, but only the lexical nodes in the target language are selected, with easier activation for the most dominant language^17^. Green proposed a third model, the inhibitory control model, under which there is activation of concepts in both languages, but with one language being suppressed via an inhibitory mechanism^18^. This model explains a further empirical observation: there is a larger cost associated with switching from a non-dominant language back to a dominant language than the other way around. Under the inhibitory control model, when producing your non-dominant language, more inhibition of the dominant language is required, thus leading to slower responses when returning to the dominant language^19^. The language-specific selection model and inhibitory control model differ on the existence of a competitive selection process in selecting the correct target word: the inhibitory control model assumes competition among words in the non-target language that need to be suppressed via inhibition, while no such process is assumed in the language-specific selection model.

Critically, in both models, the notion of proficiency is not well-defined. Participants in studies that test these models define proficiency either by self-reported expertise or age of acquisition, where earlier acquisition equates with higher proficiency. These two measures are typically highly correlated^20,21^, although there are instances where they are disentangled, with speakers unable to speak a language acquired early^22^, and speakers becoming fluent in a language acquired later^23^. In these models, lexical selection is proposed to be easier in the more proficient language, and similar for equally proficient languages^7^. Critically, proficiency seems to be assumed to be consistent across the two languages for all categories.

Differences in lexical access have not only been observed between the two languages of a bilingual speaker, but several studies also report differences in bilingual and monolingual lexical access. First, bilinguals take longer than monolinguals in naming pictures and producing noun phrases^24–26^. Second, this difference is exacerbated for lower frequency words^27^. And third, the difference disappears with words with equal frequencies, such as proper nouns^28^, or where words between the two languages are cognate words^5,29^.

Consequently, Gollan et al. proposed the frequency-lag hypothesis^13,24,25^, such that bilinguals tend to be exposed to words in each of their languages less than a monolingual (in one language), thus leading to slower access by the bilingual to the lexemes of their two languages. Blanco-Elorrieta and Caramazza incorporate this notion into their model, where frequency of a word is used to explain lexical access effects, but not an independent notion of proficiency (as in the previous models)^30^. This proposal thus dispenses with the notion of overall proficiency as an explanatory factor, in favor of exposure frequency alone. Exposure frequency, in turn, does not assume uniformity across categories of a language: one can be more exposed for some words in one language, and for other words in another language.

A strong prediction of the category-specific proficiency hypothesis is that it should be possible to find conceptual domains where words are more frequent in the globally less proficient language. Such examples have not been investigated thus far because the languages that have been investigated so far come from similar cultures, and hence the frequencies of related words were highly correlated^31^.

In the current study, we rely on the vast cultural differences between industrialized cultures and non-industrialized cultures to investigate this prediction, leveraging the benefits of studying non-Western, Educated, Industrialized, Rich and Democratic (i.e., non-WEIRD) societies^32^. Specifically, we look at the domain of color concepts, which are labeled more frequently in industrialized cultures than non-industrialized cultures^33^. One such example is Tsimane’–Spanish bilinguals. Tsimane’ speakers are farmer–foragers living in the Bolivian Amazon rainforest^34^; in contrast to industrialized cultures, the Tsimane’ do not talk about color very much and consequently have a limited shared color vocabulary^33^. Whereas all Tsimane’ speakers know words corresponding to black (*tsincus*), white (*jaibes*) and red (*jaines*), monolingual Tsimane’ speakers use less consistent labels for other colors. However, due to globalization, some Tsimane’ leave their communities temporarily to visit nearby Spanish-speaking villages and thus end up learning Spanish as well. In Bolivian Spanish, there are 12 commonly used color words: *blanco* (white), *negro* (black), rojo (red), *verde* (green), *amarillo* (yellow), *celeste* (light blue), *azul* (dark blue), *rosado* (pink), *anaranjado* (orange), *violeta* (purple), and *gris* (grey). Spanish speakers show greater consistency of labeling for most colors, compared to Tsimane’. Furthermore, Spanish-Tsimane’ bilinguals show greater consistency in labeling most colors in Tsimane’ than Tsimane’ monolinguals (Malik-Moraleda et al., in prep.). Thus, although Tsimane’ monolinguals have a few consistent color labels, Tsimane’–Spanish bilinguals have existing Tsimane’ labels for the color words *tsincus* (black), *jaibes* (white), *jaines* (red), *shandyes* (green), *chames* (yellow), *nyushnus* (blue), *chocolateyeisi* or *cafeyeisi* (brown), *itsiyeisi* (purple), *chimdyes* (grey). The consistency of labeling is plausibly an indirect measure of frequency of use across individuals^33^.

Our critical task is lexical access via picture-description, where a Tsimane’–Spanish bilingual participant is asked to name a color or animal as fast as possible, in the target language (Tsimane’ or Spanish). For the control set of materials—animals—both theories, i.e., the overall-proficiency and category-specific proficiency theories, predict faster and more accurate naming in Tsimane’ than Spanish for these Tsimane’–Spanish bilinguals, because these are common local animals, which the Tsimane’ know and have talked about in Tsimane’. For both the overall proficiency and category-specific proficiency-based theories, this follows because the speakers are more proficient in Tsimane’ and hence they should access words in Tsimane’ more easily.

The theories make differing predictions for the color-labeling task. The overall-proficiency-based theories make the same prediction as for the animals: faster and more accurate naming in Tsimane’ than Spanish, because the participants are more proficient in Tsimane’. On the other hand, the category-specific proficiency-based theory relies only on word frequency to explain lexical access, and hence predicts faster and more accurate labeling in Spanish than Tsimane’ overall, because color words are more frequent in Spanish than Tsimane’, and hence the Tsimane’–Spanish bilinguals will probably have been exposed to the Spanish words more overall than the Tsimane’ versions. In other words, the category-specific proficiency-based theory predicts an interaction between language and word-type (i.e., animals and colors), while the overall proficiency-based theory predicts only a main effect of language.

Furthermore, if frequency is the main driver in proficiency, the ease of access should depend on the particular color words. The only Tsimane’ color terms that all speakers share are *jaibes* (white), *tsincus* (black) and *jaines* (red). It is plausible that the frequency of these words is larger than their Spanish counterparts, in the experience of the Tsimane’–Spanish bilinguals. Thus, it is possible that the Spanish dominance may apply only to the other color words that we tested—*yushnus/azul* (blue), *chocolateyeisi/marrón* (brown), *shandyes/verde* (green), *chimdyes/gris* (grey), *chames/amarillo* (yellow)– where the Spanish words are likely much more frequent than the Tsimane’ counterparts.

## Results

Given that Tsimane’ monolingual speakers have idiosyncratic responses for less frequent color words^33^, and tend to use one term for all of green and blue (‘grue’)^33,35^, we first sought to ensure that the conceptual representation for each of the tested color words was similar for the two languages.

**Figure 1:**
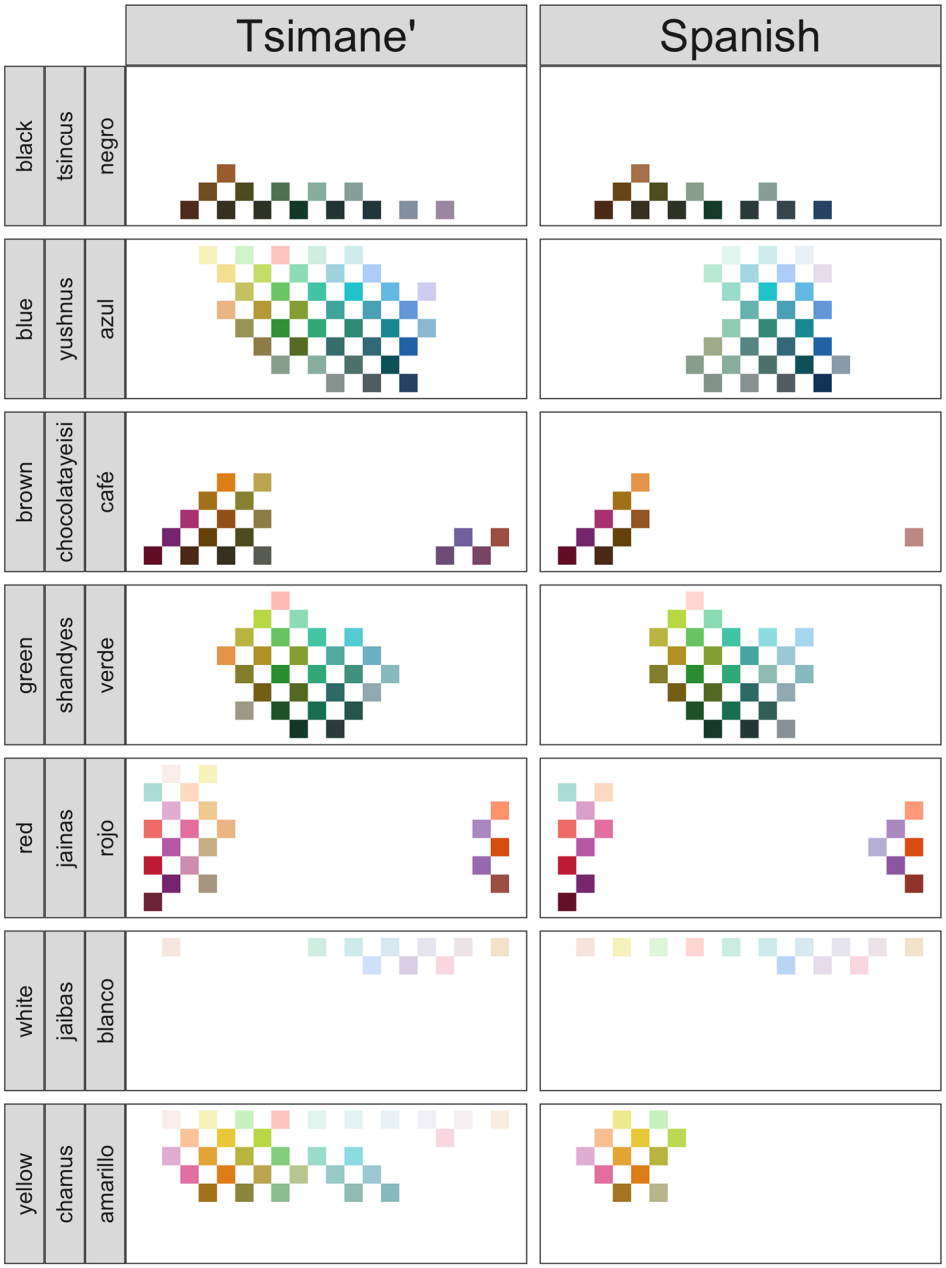
Representation of colors by Tsimane’–Spanish bilinguals in Tsimane’ and Spanish for seven of the eight color terms tested in the naming task. All chips colored in the graph correspond to chips chosen during the labeling task for the term indicated on the y axis. The terms were asked in the language indicated on the column label, and the most common label for that color term in Tsimane’ and Spanish (as well as the English equivalent, for presentation purposes), is given in the row labels, but see Supp. Table 2 for a full list of the existing terms in Tsimane’ for each of the colors. The eighth color term—“gray”/“chimdyes”/“gris”—was not investigated using this task, because there are no good exemplars of this color term on the surface of the Munsell color sphere.

First, we examined whether the distribution of the chips selected in the Munsell chips labeling task for each of the color terms was similar. As can be observed in Fig. 1, the chips chosen in the task were similar between Tsimane’ and Spanish for five of the seven colors: tsincus/negro (X^2^ = 13.51, df = 16, p = 0.63), green (Χ^2^ = 12.26, df = 28, p = 0.99), red (Χ^2^ = 12.61, df = 23, p = 0.96), white (Χ^2^ = 5.30, df = 13, p = 0.97) and yellow (Χ^2^ = 34.78, df = 34, p = 0.43). The distributions for the remaining two colors that we tested differed (blue: Χ^2^ = 70.6, df = 43, p = 0.005; brown: Χ^2^ = 28.033, df = 17, p = 0.04).

Second, we asked whether the mean hue values of chips chosen for each of the color words fell within the canonical ranges established by the ISCC-NBS System^36^. which was created by the Inter-Society Color Council, a group of vision scientists^37^. The hue of a color is the attribute that distinguishes one color from another. In the Munsell space, hues are placed in a circle, with red, yellow, green, blue, and purple placed at approximately equal intervals around the circle. We obtained the hue values for each of the chips in our array with the R package ‘munsellinterpol v.2.6-1’ from Gama et al.^36^. In the Munsell array used in the present experiment, hue is represented across columns, with the hue in column 1 being 5, the hue value of column 10 is 50, and the hue value of column 20 being 100. According to the ISCC-NBS System, the range of hue values from 55 (green–blue) to 75 (purple–blue) is “blue”; from 10 (red–brown) to 25 (yellow–brown) is “brown”; from 35 (green–yellow) to 55 (green–blue) is “green”; from 5 (red) to 95 (purple–red) is “red”; and from 15 (yellow–red) to 35 (green–yellow) is “yellow”. The mean hues of the chips chosen by speakers to represent the terms nyushnyes/azul (“blue”; 58.1 in Tsimane’ and 66.8 in Spanish), chocolateyeisi/café (“brown”; 21.3 in Tsimane’ and 16.0 in Spanish), shandyes/verde (“green”; 42.6 in Tsimane’ and 43.0 in Spanish), jainas/rojo (“red”; 17.0 in Tsimane’ and 18.7 in Spanish) and chames/amarillo (“yellow”; 30.2 in Tsimane’ and 24.2 in Spanish) fell within the established range, thus indicating that all these terms fall within conventional boundaries for each of the color terms. The colors black, white, and grey do not have hue values, and participants were not asked about the color grey.

Taken together, these results suggest that there are similar color concepts underlying the Tsimane’ and Spanish color terms that we investigated.

Given that the representations of the color terms in the two languages were similar, we proceeded to look at accuracy and reaction time data in the naming task. The two predictor variables we were interested in were overall proficiency and category-specific proficiency. As established in the introduction, category-specific is captured by *word-type* (animals, colors), as animal words are similarly frequent in Spanish and Tsimane’, while color words are more frequent in Spanish than Tsimane’. On the other hand, overall proficiency was captured by the variable *language*, as all participants rated themselves to be more proficient in Tsimane’ than Spanish; this was established by asking participants to rate themselves on various dimensions (see Table 6) such as age of acquisition, percentage of use of each language, self-rated proficiency in speaking and listening, and years of immersion in a region where that language is dominant. For all 21 of the 22 participants for whom we had this information, participants rated themselves to have acquired Tsimane’ earlier (t(20) = 12.58, p < 0.01), use it more (t(20) = − 7.40, p < 0.01), and have better self-rated speaking (t(20) = 8.91 p < 0.01) and listening proficiency (t(20) = 7.62, p < 0.01), and reported spending more time in Tsimane’-speaking communities (t(19) = − 4.41, p < 0.01; the information for one of the participants on this measure was missing). Taken together, these results indicate that all the Tsimane’–Spanish bilingual speakers that took part in our study were more proficient in Tsimane’ than Spanish.

To disentangle whether overall proficiency in a language or frequency of exposure to individualized words predict better behavioral results (accuracy in naming or reaction time data), we used linear-mixed effect models for reaction time data and generalized mixed effects models for accuracy data with the R package ‘lme4’; p-value approximations was done using R package ‘rstatix’^38,39^. The models included word-type, language, and their interaction. Effects were contrast-coded [− 0.5, 0.5], except for runs, which was coded with 8 levels, one for each run. We also included trial-type (switch, non-switch) and run (from 1 to 8) as main effects. Following Meuter and Allport^19^, trials where participants switch between languages were expected to be slower and less accurate than trials where participants continue naming in the same language (i.e., non-switch trials). Furthermore, participants were expected to become faster and more accurate as they progress through the runs. Finally, subjects (n = 22) and items (n = 16; 8 color items and 8 animal items) were entered as random effects. The maximal models we started with included Word-Type × Language main effect and interactions for both subjects and items. We removed interactions and then main effects from item first and then subjects until the model converged. We report the maximal model that converged for each of the analyses below.

$$Accuracy \sim \, Word{\text{-}}type \times Language \, + \, Run \, + \, Trial{\text{-}}type \, + \, \left( {Language \, | \, Subject} \right) \, + \, \left( {1|Item} \right)$$

**Figure 2:**
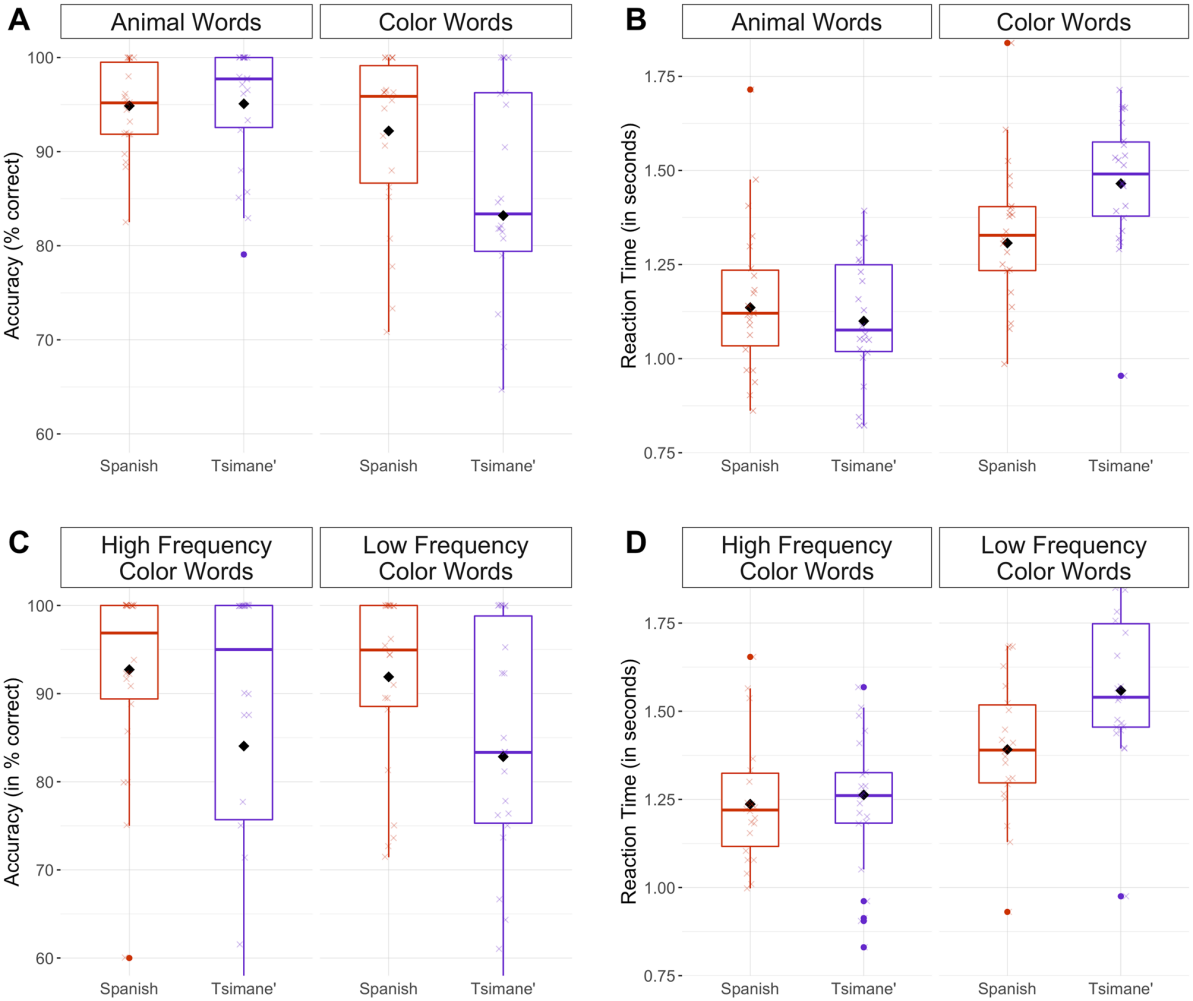
Tsimane'–Spanish bilinguals are faster at naming color words in Spanish than Tsimane', but only for low frequency color words. Figures depict accuracy (**A**) and reaction time (**B**) data in naming animal and color words in Tsimane' and Spanish, and accuracy (**C**) and reaction time (**D**) data in naming high and low frequency color words in Tsimane' and Spanish. In all subpanels, boxplot figures include median, interquartile range, standard deviation and outlier values (solid dots) for responses to each word-type in the Tsimane’–Spanish bilinguals’ two languages. The average response of each individual is depicted as crosses, jittered for visualization purposes. The mean average response across all speakers is depicted as a solid black diamond.

Results from the generalized linear mixed effect model that predicted naming accuracy (Table 1) indicated that, as expected, participants were less accurate during trials where they had to switch from one language to another (p < 0.01), as opposed to trials where they continued naming in the same language as the previous trial. Crucially, we found an interaction between the word-type of the picture (i.e., whether the participant was naming a color or an animal) and the language they were naming it in. Participants were more accurate (Fig. 2A) in naming color words in Spanish, although they were less proficient in this language.

**Table 1:** Results from the generalized linear mixed effect model predicting accuracy. Asterisks indicate main effects that reached significance.

	Estimate	Std. error	Z value	Pr(>|t|)	
(Intercept)	2.55	0.31	8.20	< 0.001	***
Word-type	1.01	0.15	− 6.65	< 0.001	***
Language	0.03	0.29	0.12	0.91	
Run	0.12	0.09	1.37	0.17	
Trial-type (switch, non-switch)	1.28	0.15	− 8.70	< 2e−16	***
Word-type × Language	− 1.12	0.30	− 3.80	< 0.001	***

What was the nature of the errors? Most of the errors came from intra-language interference (i.e., naming a different animal or color in the language cued) for both animals (86% of errors) and colors (64% of errors), although interlanguage interference (naming the correct animal or color in the wrong language) occurred more for colors (34% of errors) than for animals (11%; see Table 2 for summary of all types of errors).

$$Reaction \, Time \sim \, Word{\text{-}}type \times Language \, + \, Run \, + \, Trial{\text{-}}type \, + \, \left( {Word{\text{-}}type \times Language \, |Subject} \right) \, + \, \left( {1|Item} \right)$$

**Table 2:** Type of errors produced. Errors from within each word-type sum up to 100%.

Word-type	Type of errors	Percent
Animal	Hesitation	2.97
Animal	Inter-language	86.14
Animal	Intra-language	10.89
Color	Hesitation	0.78
Color	Inter-language	64.34
Color	Intra-language	34.88

The results from the linear mixed effect model that predicted reaction time (Table 3) followed a similar pattern. Participants were faster during trials where they did not switch between languages (p < 0.01), and faster through the runs (p = 0.02), getting better at the task as the experiment progressed. A significant interaction between word-type and language was obtained (Fig. 2B), whereby participants were equally fast when naming animals in Tsimane’ than Spanish, but faster when naming colors in Spanish than Tsimane’.

**Table 3:** Results from the linear mixed effect model predicting reaction time. Asterisks indicate main effects that reached significance.

	Estimate	Std. error	df	t value	Pr(>|t|)	
(Intercept)	1.34	0.05	43.73	26.54	< 2e−16	***
Word-type	0.28	0.07	18.70	3.87	< 0.001	**
Language	0.03	0.03	22.73	0.86	0.40	
Run	− 0.02	0.01	3049.00	− 2.15	0.03	*
Trial-type (switch, non-switch)	0.09	0.02	3037.00	5.72	< 0.001	***
Word-type × language	0.14	0.06	18.95	2.57	0.02	*

In summary, participants were better (i.e., faster and more accurate) in Spanish when naming color words despite it being the less proficient language. These results suggest that exposure to a word’s frequency drives lexical access, not overall proficiency within a language.

Are participants aware that they are faster at naming color words in Spanish than Tsimane’? After they completed the experiment, a subset of participants (n = 16) were asked whether they spoke about color words more in Tsimane’ or in Spanish. Most participants (9/16) claimed they spoke about colors more in Tsimane’, while some claimed they mainly used Spanish words (5/16) and a minority claimed they used both languages (2/16). We analyzed the data of the subjects who claimed to speak about colors primarily in Tsimane’ (n = 9; see Fig. S3, Tables S3, S4), and numerically, participants were still more accurate and faster in naming color words in Spanish (accuracy = 89.9%; RT = 1.36 s) than Tsimane’ (accuracy = 77.5%; RT = 1.46 s) as opposed to being equally accurate and fast in naming animal words in Tsimane’ (accuracy = 92.8%; RT = 1.17 s) and Spanish (accuracy = 94.6%; RT = 1.15 s). The category × language interaction was statistically reliable in this group of participants for accuracy data (beta estimate = − 0.89, p = 0.034; see Fig. S3 for visualization, Table S3 for full results of the lme model), but not for RT data (beta estimate = 0.057, p = 0.42; see Fig. S3 for visualization, Table S4 for statistical results of the lme model).

If the word-type × language interaction is driven by the fact that color words are used less often in Tsimane’ than Spanish, we would expect to find less of a difference in color naming for the color words that are most widely used in Tsimane’ (i.e., *jaibes* ~ = white, *tsincus* ~ = black, *jaines* ~ = red; *26*). To test this, a new linear mixed effect model was created to fit data for color words. We don’t have word-based frequency estimates in Tsimane’ because there are no Tsimane’ corpora yet. We therefore divide the color words into two groups: *jaibes* (white), *tsincus* (black) and *jaines* (red) trials were coded as high frequency color words and the other color words were coded as low frequency color words. The language, the frequent (high, low) and their interaction were entered in the regression, along with run (1–8) and trial-type (switch or non-switch). Finally, participants (n = 22) and trials (n = 8) were entered as random effects.

$$Accuracy \, \sim \, Frequency \times Language \, + \, Run \, + \, Trial{\text{-}}type \, + \, \left( {Language|Subject} \right) \, + \, \left( {1| \, Item} \right)$$

As expected, the results (Table 4) revealed participants’ accuracies increased as the experiment progressed (p < 0.01) and were higher for trials where they were not asked to switch between languages than for those they did (p < 0.01). No significant frequency × language interaction (p = 0.95) was observed (Fig. 2C; see Fig. S1 to see the results for each color term individually).

$$Reaction \, Time \, \sim \, Frequency \times Language \, + \, Run \, + \, Trial{\text{-}}type \, + \, \left( {Frequency \times Language \, | \, Subject} \right) \, + \, \left( {Language| \, Item} \right)$$

**Table 4:** Results from generalized linear mixed effect models predicting accuracy for color data.

	Estimate	Std. error	Z value	Pr(>|t|)	
(Intercept)	3.12	0.57	5.50	0.00	***
Frequency	− 0.02	0.31	− 0.06	0.95	
Language	− 0.86	0.50	− 1.72	0.09	
Run	0.04	0.03	1.38	0.17	
Trial-type (switch, non-switch)	− 1.37	0.05	− 28.73	< 2e−16	***
Frequency × language	0.01	0.11	0.06	0.95	

The results for reaction time data revealed a different pattern (Table 5), with participants becoming faster across runs, as well as being faster in trials where they continued naming in a language, as opposed to trials where they switched languages. Crucially, a significant frequency × language interaction was observed (p < 0.001), whereby participants were similarly fast in Tsimane’ and Spanish when naming color words that are high frequency Tsimane’ color words, but faster in Spanish when naming color words that are low frequency in Tsimane’ (Fig. 2D; see Fig. S2 to see the results for each color term individually).

**Table 5:** Results from linear mixed effect models predicting reaction time for color data.

	Estimate	Std. error	df	t value	Pr(>|t|)	
(Intercept)	1.39	0.10	10.85	14.61	< 0.001	***
Frequency	0.21	0.12	9.67	1.83	0.10	
Language	− 0.03	0.07	19.88	− 0.49	0.63	
Run	− 0.04	< 0.001	23,060.00	− 12.67	< 2e−16	***
Trial-type (switch, non-switch)	0.05	0.01	22,810.00	8.40	< 2e−16	***
Frequency × language	0.20	0.08	15.28	2.50	0.02	*

## Discussion

We sought to understand whether overall proficiency in a language (as defined by self-report) or category-proficiency (as measured by frequency of exposure to words) is more important in bilingual lexical access by probing Tsimane’–Spanish bilinguals in a naming task. While frequency of a word and overall proficiency of a language are usually confounded, Tsimane’–Spanish bilinguals encounter color terms more frequently in their less proficient Spanish than in their native Tsimane’, therefore allowing for the disentangling of the two factors. First, we observed that the bilingual speakers’ understanding of color was not conceptually different between their two languages. Critically, results in a picture-naming task indicated that, despite reporting to be overall more proficient in Tsimane’, Tsimane’–Spanish bilinguals were better at naming colors in Spanish, except for the three color terms that are of higher frequency in Tsimane’ (i.e., *jaibes* ~ = ‘white’, *tsincus* ~ = ‘black’, *jaines* ~ = ‘red’). These results seem to indicate that category-specific proficiency is a more important factor than overall proficiency in accessing words. In other words, lexical access depends on exposure to words, irrespective of the overall proficiency in the language.

Of all four bilingual lexical access models in the literature, only Blanco-Elorrieta and Caramazza’s ‘Common selection mechanism’ predicted that lexical access would be easier in Spanish for Tsimane’–Spanish bilinguals for color words. The other three models do not explicitly accommodate our results. First, La Heij’s model does not predict the inter-language interference we observe during the picture naming task (Table 2), as it argues that bilinguals’ lexical nodes are activated at the preverbal level and only words in the target language would be activated. Second and third, Costa’s and Green’s models can only accommodate the results if proficiency is defined to be as category-specific, as opposed to overall proficiency in a language, as it has been probed in the past. In other words, both models would need to define better what they mean by proficiency. In the past, proficiency has assumed to always refer to *overall* proficiency; therefore both the accuracy and reaction time data reported here for animals are predicted by these models, but, according to these models, the results for color words should have followed a similar pattern, which is not the case.

The importance of frequency in lexical access has already been established in monolingual speakers^40–43^, and reported in bilingual speakers^24,25^. However, in bilingual speakers the frequency effect had been reported within each language separately, and with higher overall frequencies for the more proficient language, while our current results suggests that the effect transcends the two languages, i.e., the frequency of a word is not necessarily higher for the most proficient language and needs to be considered separately from overall proficiency of a language.

Two interpretations of the results are possible: a strong version would argue that category-specific proficiency is, in fact, explained by frequency. A second interpretation possible is that there might be two factors at play in lexical access: overall proficiency of a speaker in a language, and ease of access to certain domains based on our experience. While here we establish that of the two factors (overall proficiency in a language, and category-specific proficiency, i.e., word exposure measured by frequency), word exposure plays a larger role in lexical access; future research may be able to explore whether overall proficiency in a language also plays a significant role in lexical access as well, or if it’s entirely driven by word exposure.

While the present study is an example of why the study of under-represented, non-WEIRD communities can benefit our understanding of language processing in particular and cognitive processing in general, our results are not necessarily restricted to these populations. Specifically, the dissociation between frequency and proficiency is not confined to cases of farmer–forager tribes becoming more industrialized but might be more common in bilingual settings of industrialized societies too. For instance, a non-native English speaker attending an English-based university will encounter academic terminology in their non-dominant language^14^. It so happens that in the Tsimane’, this effect occurs at the population level, while in the other case discussed above, the effect is idiosyncratic to speakers and their experiences. In other language pairs, unlike in Tsimane’, which does not have a corpus, larger corpora can be used to detect differences between the frequencies of translation word pairs. An interesting scenario may be languages where new terminology and translations are being introduced, as is the case for scientific terminology^44^.

## Methods

### Participants

Twenty-two Tsimane’–Spanish bilinguals took part in the experiment. All bilinguals first learned Tsimane’ and then Spanish, and all participants rated themselves to be more proficient in Tsimane’ than Spanish (see Table 6 for details; NB only information for 21 participants were obtained for the proficiency measures). Participants were screened for color-blindness^45^ prior to the study, received compensation for their time, gave an informed consent as required by the Committee on the Use of Humans as Experimental Subjects (COUHES) from MIT and were paid for their participation. The sample size was determined by convenience, given the limited number of participants available for testing.

**Table 6:** Linguistic profile of the bilingual population. Participants were asked to rate their proficiency from 1 (excellent) to 5 (beginner). Years of immersion refers to the amount of time the speaker has lived in a community where the dominant language is either Tsimane’ or Spanish. SD in brackets.

Language	Age of acquisition	Percentage of use	Self-rated proficiency		Years of immersion
			Speaking	Listening	
Tsimane’	2.38 (1.56)	73.8 (14.4)	1.05 (0.2)	1.12 (0.3)	10.5 (2.67)
Spanish	12.6 (3.43)	26.2 (14.4)	2.71 (0.8)	2.90 (0.9)	8.49 (1.04)

### Tasks

Participants took part in two separate tasks: Munsell chips labeling, and a picture naming task.

In the *Munsell chips labeling* task, participants were asked to name 84 colored chips sampled from the standard Munsell array of colors and presented to them in a random order (see Table S1 for the Munsell chips used in the experiment and^46^ for an introduction to the Munsell color system). Participants were asked to label the chips in Tsimane’ first, and then in Spanish, or vice versa (see Table S2 for all the labels used by participants). This task was performed indoors under controlled lighting conditions with the use of a light box (nine phosphor broadband D50 color-viewing system, model PDV-e, GTI Graphic Technology, Inc.).

In the *picture naming* task, participants were asked to name 8 colors (tsincus/negro = English ‘black’, yushnus/azul = English ‘blue’, chocolateyeisi/café = English ‘brown’, shandyes/verde = English ‘green’, chimdyes/gris = English ‘grey’, jainas/rojo = English ‘red’, chames/amarillo = English ‘yellow’, jaibas/blanco = English ‘white’) and 8 animals (batatak/mariposa = English ‘butterfly’, acho/perro = English ‘dog’, tabelle/pescado = English ‘fish’, okoko/rana = English ‘frog’, oyo/mono = English ‘monkey’, nas/bivora = English ‘snake’, otoko/sama = English ‘spider’, shi/anta = English ‘tapir’) in Tsimane’ and in Spanish. Given the heterogeneity across monolingual Tsimane’ speakers in naming colors^33^, participants were first shown individual pictures of the colors and the animals, and their labels for each were annotated in order to allow for corrections. Then, participants were presented with a series of cued trials, consisting of a fixation cross at the center of the screen presented for 700 ms, followed by a cue presented for 300 ms, followed by the target for a total of 3000 ms. Participants were asked to name the target in either Tsimane’ (cued by a Bolivian flag; cues were chosen after discussing with Tsimane’–Spanish translators) or Spanish (cued by a Spanish flag); once the target was named, the target disappeared, and a fixation cross would appear until the 3000 ms elapsed. Participants labeled both colors and animals for a total of 400 trials, alternating between Spanish and Tsimane’ trials at random intervals for 150 of the 400 trials. Due to the length of the experiment, participants were asked to name 50 trials at a time, with each set of 50 trials forming a run, for a total of 8 runs. A break was provided between runs, with the participant deciding on the duration of the break; they continued to the next run by pressing the space key on the keyboard. All stimuli were presented using Psychtoolbox^47,48^. Methods were carried out in accordance with relevant guidelines and regulations by the Committee on the Use of Humans as Experimental Subjects (COUHES) from MIT. All experimental protocols were approved by the Tsimane' Council in San Borja and the Centro Boliviano de Desarrollo Socio Integral (CBIDSI).

## Supplementary Information

Supplementary Information.

## Data Availability

All data, code, and materials used in the analyses are available in the following OSF repository: https://osf.io/5z269/.
