# Supplementary material for: Color naming in Tsimane’–Spanish bilinguals indicates that differential experience with content domains affects lexical access
Source: Sci Rep. 2022 Oct 19;12:17479. doi: 10.1038/s41598-022-18461-9 (PMC9582210; doi:10.1038/s41598-022-18461-9)
Supplement: Supplementary file 1 — Supplementary Information. [file 41598_2022_18461_MOESM1_ESM.pdf]

Supplementary Information for Malik-Moraleda & Gibson (2022)

grid_location	Munsell Code	Hue	Value	Chroma
A1	5R 9/2	5	9	2
A2	10R 9/2	10	9	2
A3	5YR 9/2	15	9	2
A4	10YR 9/4	20	9	4
A5	5Y 9/6	25	9	6
A6	10Y 9/6	30	9	6
A7	5GY 9/4	35	9	4
A8	10GY 9/4	40	9	4
A9	5G 9/2	45	9	2
A10	10G 9/2	50	9	2
A11	5BG 9/2	55	9	2
A12	10BG 9/2	60	9	2
A13	5B 9/2	65	9	2
A14	10B 9/2	70	9	2
A15	5PB 9/2	75	9	2
A16	10PB 9/2	80	9	2
A17	5P 9/2	85	9	2
A18	10P 9/2	90	9	2
A19	5RP 9/2	95	9	2
A20	10RP 9/2	100	9	2
B1	5R 8/6	5	8	6
B2	10R 8/6	10	8	6
B3	5YR 8/8	15	8	8
B4	10YR 8/14	20	8	14
B5	5Y 8/14	25	8	14
B6	10Y 8/12	30	8	12
B7	5GY 8/10	35	8	10
B8	10GY 8/8	40	8	8

B9	5G 8/6	45	8	6
B10	10G 8/6	50	8	6
B11	5BG 8/4	55	8	4
B12	10BG 8/4	60	8	4
B13	5B 8/4	65	8	4
B14	10B 8/6	70	8	6
B15	5PB 8/6	75	8	6
B16	10PB 8/4	80	8	4
B17	5P 8/4	85	8	4
B18	10P 8/6	90	8	6
B19	5RP 8/6	95	8	6
B20	10RP 8/6	100	8	6
C1	5R 7/10	5	7	10
C2	10R 7/10	10	7	10
C3	5YR 7/14	15	7	14
C4	10YR 7/14	20	7	14
C5	5Y 7/12	25	7	12
C6	10Y 7/12	30	7	12
C7	5GY 7/12	35	7	12
C8	10GY 7/10	40	7	10
C9	5G 7/10	45	7	10
C10	10G 7/8	50	7	8
C11	5BG 7/8	55	7	8
C12	10BG 7/8	60	7	8
C13	5B 7/8	65	7	8
C14	10B 7/8	70	7	8
C15	5PB 7/8	75	7	8
C16	10PB 7/8	80	7	8
C17	5P 7/8	85	7	8
C18	10P 7/8	90	7	8

C19	5RP 7/10	95	7	10
C20	10RP 7/8	100	7	8
D1	5R 6/12	5	6	12
D2	10R 6/14	10	6	14
D3	5YR 6/14	15	6	14
D4	10YR 6/12	20	6	12
D5	5Y 6/10	25	6	10
D6	10Y 6/10	30	6	10
D7	5GY 6/10	35	6	10
D8	10GY 6/12	40	6	12
D9	5G 6/10	45	6	10
D10	10G 6/10	50	6	10
D11	5BG 6/10	55	6	10
D12	10BG 6/8	60	6	8
D13	5B 6/10	65	6	10
D14	10B 6/10	70	6	10
D15	5PB 6/10	75	6	10
D16	10PB 6/10	80	6	10
D17	5P 6/8	85	6	8
D18	10P 6/10	90	6	10
D19	5RP 6/12	95	6	12
D20	10RP 6/12	100	6	12
E1	5R 5/14	5	5	14
E2	10R 5/16	10	5	16
E3	5YR 5/12	15	5	12
E4	10YR 5/10	20	5	10
E5	5Y 5/8	25	5	8
E6	10Y 5/8	30	5	8
E7	5GY 5/10	35	5	10
E8	10GY 5/12	40	5	12

E9	5G 5/10	45	5	10
E10	10G 5/10	50	5	10
E11	5BG 5/10	55	5	10
E12	10BG 5/10	60	5	10
E13	5B 5/10	65	5	10
E14	10B 5/12	70	5	12
E15	5PB 5/12	75	5	12
E16	10PB 5/10	80	5	10
E17	5P 5/10	85	5	10
E18	10P 5/12	90	5	12
E19	5RP 5/12	95	5	12
E20	10RP 5/14	100	5	14
F1	5R 4/14	5	4	14
F2	10R 4/12	10	4	12
F3	5YR 4/8	15	4	8
F4	10YR 4/8	20	4	8
F5	5Y 4/6	25	4	6
F6	10Y 4/6	30	4	6
F7	5GY 4/8	35	4	8
F8	10GY 4/8	40	4	8
F9	5G 4/10	45	4	10
F10	10G 4/10	50	4	10
F11	5BG 4/8	55	4	8
F12	10BG 4/8	60	4	8
F13	5B 4/10	65	4	10
F14	10B 4/10	70	4	10
F15	5PB 4/12	75	4	12
F16	10PB 4/12	80	4	12
F17	5P 4/12	85	4	12
F18	10P 4/12	90	4	12

F19	5RP 4/12	95	4	12
F20	10RP 4/14	100	4	14
G1	5R 3/10	5	3	10
G2	10R 3/10	10	3	10
G3	5YR 3/6	15	3	6
G4	10YR 3/6	20	3	6
G5	5Y 3/4	25	3	4
G6	10Y 3/4	30	3	4
G7	5GY 3/6	35	3	6
G8	10GY 3/6	40	3	6
G9	5G 3/8	45	3	8
G10	10G 3/8	50	3	8
G11	5BG 3/8	55	3	8
G12	10BG 3/8	60	3	8
G13	5B 3/8	65	3	8
G14	10B 3/10	70	3	10
G15	5PB 3/10	75	3	10
G16	10PB 3/10	80	3	10
G17	5P 3/10	85	3	10
G18	10P 3/10	90	3	10
G19	5RP 3/10	95	3	10
G20	10RP 3/10	100	3	10
H1	5R 2/8	5	2	8
H2	10R 2/6	10	2	6
H3	5YR 2/4	15	2	4
H4	10YR 2/2	20	2	2
H5	5Y 2/2	25	2	2
H6	10Y 2/2	30	2	2
H7	5GY 2/2	35	2	2
H8	10GY 2/4	40	2	4

H9	5G 2/6	45	2	6
H10	10G 2/6	50	2	6
H11	5BG 2/6	55	2	6
H12	10BG 2/6	60	2	6
H13	5B 2/6	65	2	6
H14	10B 2/6	70	2	6
H15	5PB 2/8	75	2	8
H16	10PB 2/10	80	2	10
H17	5P 2/8	85	2	8
H18	10P 2/6	90	2	6
H19	5RP 2/8	95	2	8
H20	10RP 2/8	100	2	8

Table S1: Munsell Code for each of the chips on the Munsell array on which participants completed their tasks, along with the hue, value and chroma values of each of the chip as computed with R package ‘munsellinterpol v.2.6-1’

English Term	Tsimane' Term	Spanish Term
black	tsincus	negro
blue	yushnus	azul
brown	chocolatayeisi	café
brown	cafeyeisi	café
green	shandyes	verde
green	gojodjeisi	green
grey	chimdyes	gris
grey	butryus	gris
orange	chuinas	anaranjado
purple	itsidyeisi	morado
red	jainas	rojo
white	jaibas	blanco
yellow	cuchicuciyeisi	amarillo
yellow	vashes	amarillo
yellow	tsundyes	amarillo
yellow	ifuyeisi	amarillo
yellow	chamus	amarillo

Table S2: Color words generally used by Tsimane' monolingual speakers for different color terms.

	Estimate	*Std. Error*	*Z value*	*Pr(>|t|)*	
(Intercept)	2.91666	0.62241	4.686	2.79E-06	***
Frequency	-1.10161	0.22002	-5.007	5.53E-07	***
Language	0.39971	0.68804	0.581	0.5613	
Run	-0.03535	0.13063	-0.271	0.7867	
Trial-type (Switch, Non-Switch)	-1.08256	0.21099	-5.131	2.88E-07	***
*Frequency * Language*	-0.89227	0.42213	-2.114	0.0345	*

Table S3: Results from generalized linear mixed effect models predicting accuracy for color data in Tsimane' speakers that claimed to talk about color words more in Tsimane' than Spanish (n=9). Asterisks indicate main effects that reached significance.

	Estimate	*Std. Error*	*df*	*t value*	*Pr(>|t|)*	
(Intercept)	1.38023	0.0441	54.22134	31.301	< 2e-16	***
Word-type	0.25521	0.06392	18.03155	3.993	0.000851	***
Language	0.04817	0.03946	10.80878	1.221	0.248184	
Run	-0.03877	0.01396	1233.82024	-2.778	0.005555	**
Trial-type (Switch, Non-Switch)	0.06976	0.02428	1239.26965	2.873	0.00413	**
*Word-type * Language*	0.05769	0.06969	9.50111	0.828	0.428055	

Table S4: Results from the linear mixed effect model predicting reaction time for color data in Tsimane' speakers that claimed to talk about color words more in Tsimane' than Spanish (n=9). Asterisks indicate main effects that reached significance.

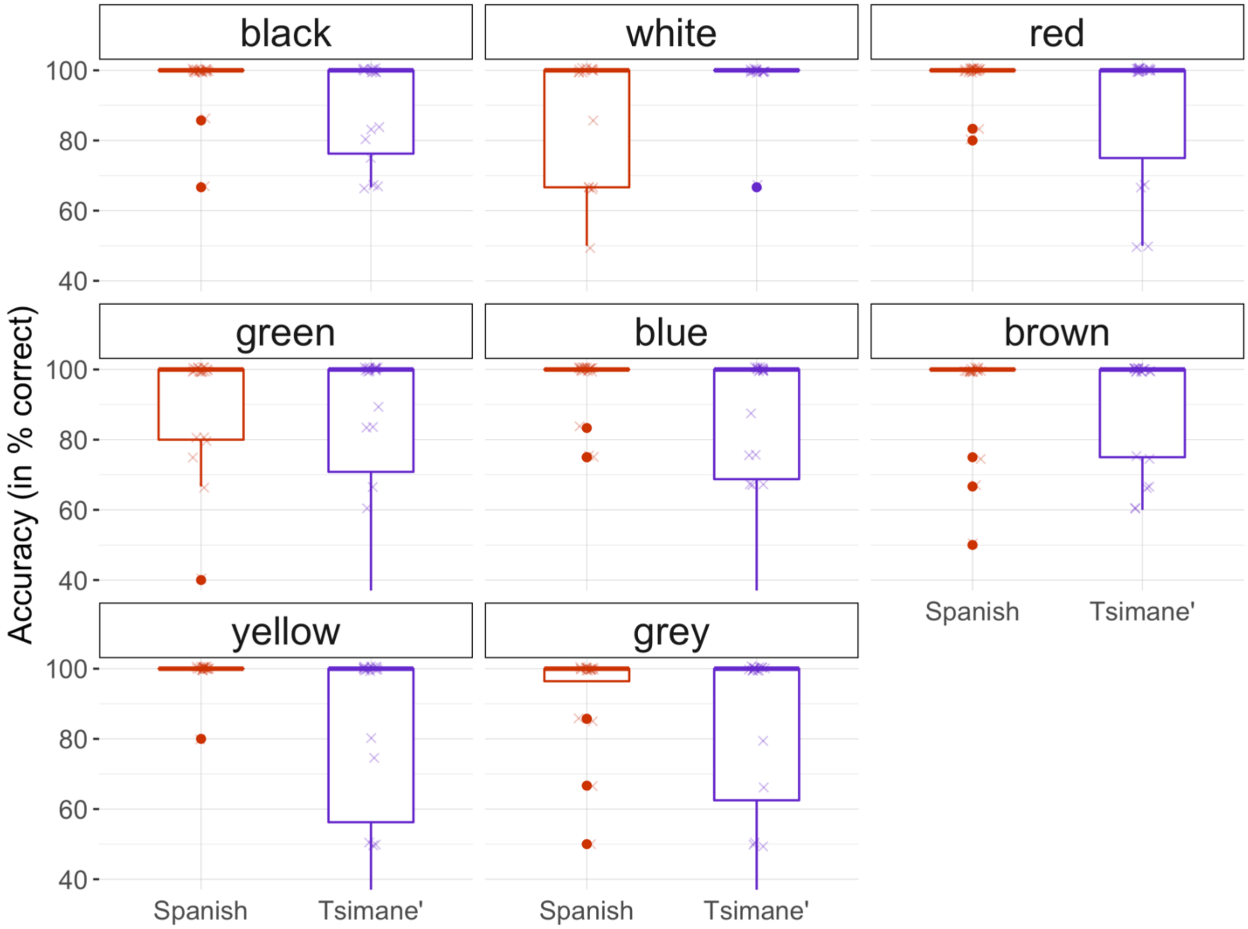

Fig. S1: Accuracy data for each of the color words tested. Boxplot figures include median, interquartile range, standard deviation, and outlier values (solid dots) for responses to each word-type in the Tsimane'-Spanish bilinguals' two languages. The average response of each individual is depicted as crosses, jittered for visualization purposes.

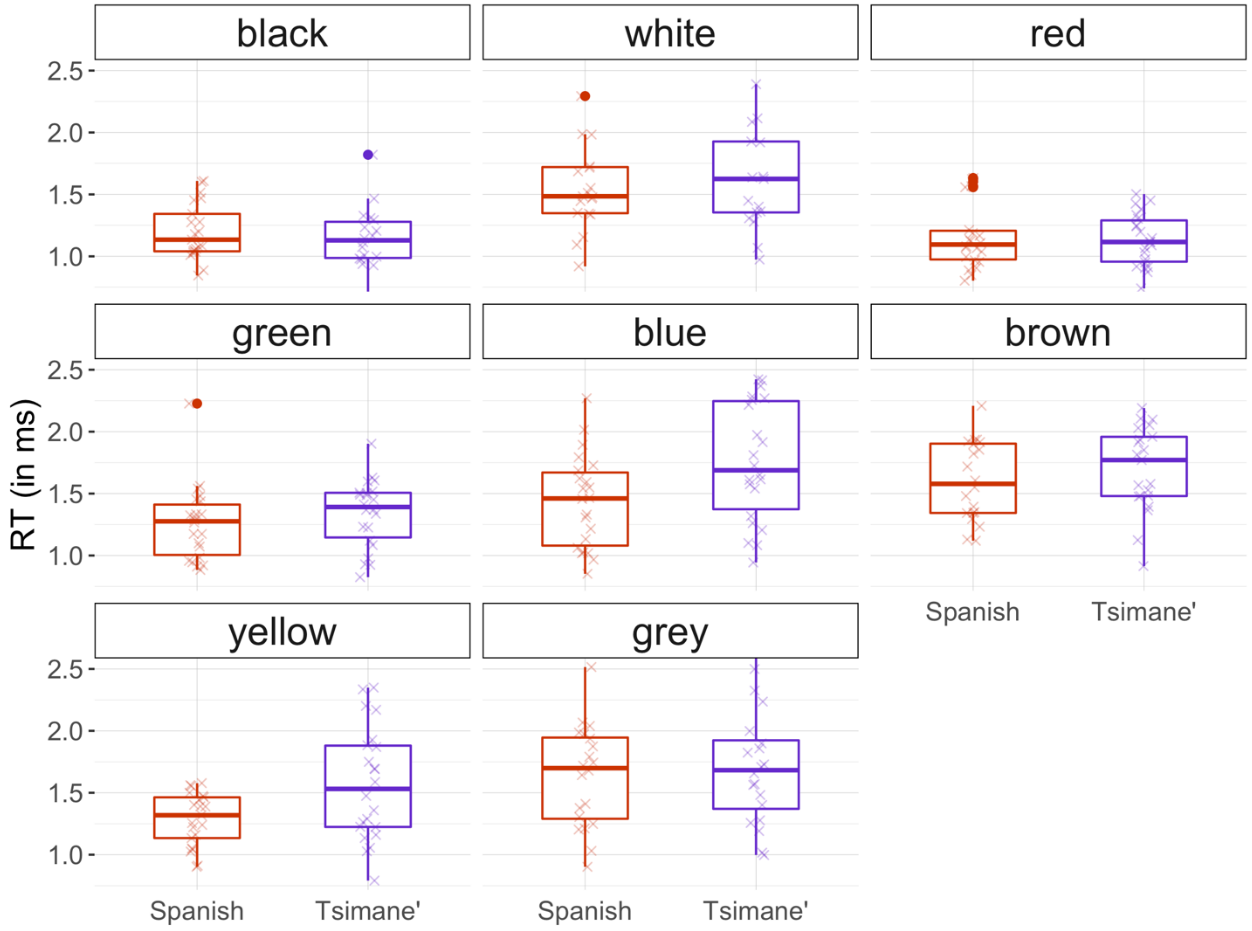

Fig. S2: RT data for each of the color words tested. Boxplot figures include median, interquartile range, standard deviation, and outlier values (solid dots) for responses to each word-type in the Tsimane'-Spanish bilinguals' two languages. The average response of each individual is depicted as crosses, jittered for visualization purposes.

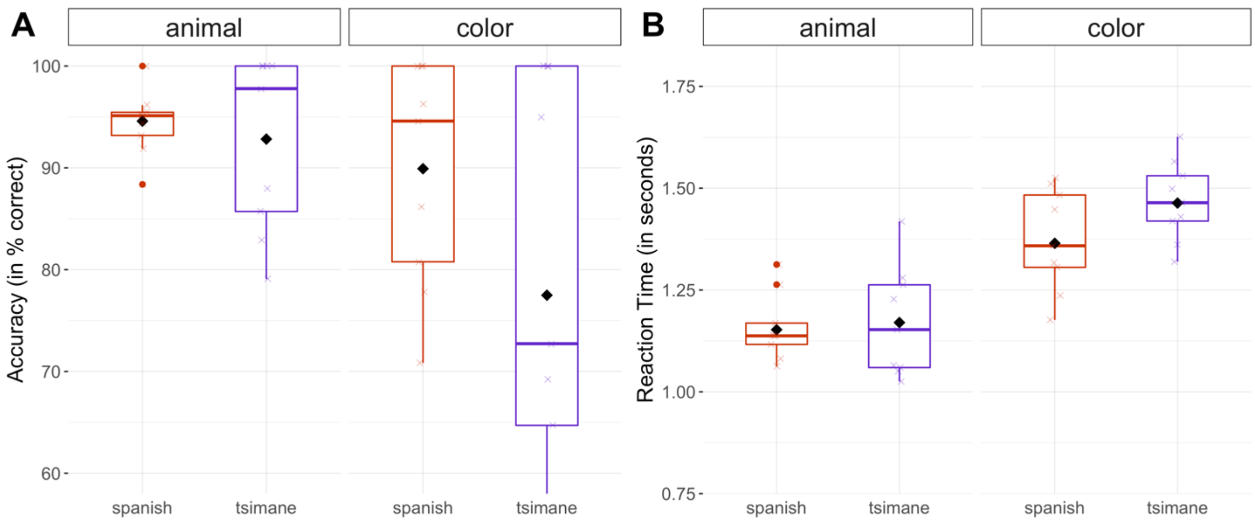

Fig. S3: Accuracy and RT data for color and animal words in subject (n=9) who, when asked whether they spoke about colors in Tsimane' or Spanish, said they spoke about color words in Tsimane'. Boxplot figures include median, interquartile range, standard deviation, and outlier values (solid dots) for responses to each word-type in the Tsimane'-Spanish bilinguals' two languages. The average response of each individual is depicted as crosses, jittered for visualization purposes.
